# Multi-Focal Giant Cell Tumor of A Single Tendon Sheath: A Rare Case Report

**DOI:** 10.7759/cureus.37600

**Published:** 2023-04-15

**Authors:** Samuel D Novick, Puneet Kahlon, Mefthe Berhanu, Parakh Patel, Muskan Uderani, Faraz Saleem

**Affiliations:** 1 General Surgery, Nassau University Medical Center, East Meadow, USA; 2 Medical School, University of Nicosia Medical School, Nicosia, CYP; 3 Internal Medicine, American International Medical University, Gros Islet, LCA; 4 Health Sciences, University of Texas Health Science Center at Houston, Houston, USA; 5 Medicine, American International Medical University, Gros Islet, LCA; 6 Internal Medicine, Liaquat University of Medical and Health Sciences, Hyderabad, PAK; 7 Internal Medicine, California Institute of Behavioral Neurosciences & Psychology, Fairfield, USA; 8 Internal Medicine, Akhtar Saeed Medical and Dental College, Lahore, PAK

**Keywords:** flp, flexor policis longus, tumor, gctts, giant cell tumor of tendon sheath

## Abstract

A giant cell tumor of the tendon sheath (GCTTS) is a rare benign tumor that typically presents as a solitary mass in the hand or wrist. Multifocal presentation of GCTTS is extremely rare and has been reported in only a few cases. Although the origin of multifocal giant cell tumors of the tendon sheath remains incompletely elucidated, it is a rare disorder that distinguishes itself from the diffuse form of GCTTS that typically occurs near major joints. In this case study, we report a patient with a localized multifocal GCTTS affecting the tendon sheath of the flexor pollicis longus (FPL) on the volar surface of the right thumb. The diagnosis was confirmed by both radiological and histological examinations. Additionally, the patient underwent surgical excision of the tumor masses and did not encounter any recurrence during the six-month follow-up period.

## Introduction

A giant cell tumor of the tendon sheath (GCTTS) is a common hand tumor, with only the ganglion cyst of the hand occurring more frequently. GCTTS tends to affect women more than men and typically presents between the ages of 30 and 40. It is also referred to as a tenosynovial giant cell tumor or pigmented villonodular tenosynovitis, and it is classified as either localized or diffuse [1,2].

The origin and underlying causes of GCTTS are still not fully understood. There are a few possible explanations, including an inflammatory response triggered by long-term exposure to antigens, a reactive overgrowth from the synovial lining of the joint and tendon sheath, or a lesion that originates from monocyte or macrophage cells [3].

GCTTS typically presents as a soft-tissue lump near a small joint in the hand or foot. The lump arises from a tendon sheath, the synovial lining of a joint, or bursa, and is usually painless and grows slowly. It may be attached to deep structures and press against the bone, leading to erosion and scalloping [4].

Histological analysis of GCTTS often shows a nodular lesion with a distinct border composed of mononuclear cells arranged diffusely alongside scattered osteoclast-like giant cells. There may also be areas with foam cell infiltration. The mononuclear cells have histiocytic morphology, featuring vesicular nuclei, small nucleoli, nuclear grooves, and cytoplasm that appears vacuolated or pale eosinophilic [1].

An MRI of the lesion commonly revealed the presence of distinct swellings originating from the tendons. The size and spread of the masses were also visualized using this imaging modality. Given the high risk of recurrence, it is recommended to remove the entire tumor to minimize the chances of recurrence. Complete local excision is the preferred treatment approach [1, 2, 3].

## Case presentation

A 34-year-old male presented to a tertiary care hospital's orthopedic department with painless and lobulated swelling over his right thumb for the past six months. The patient was having difficulty flexing his right thumb at the interphalangeal (IP) joint due to swelling over the volar aspect of his thumb and had occasional pain around the swelling, which was worse with activity. He denied any history of trauma, fever, chills, or weight loss. He tried over-the-counter analgesics, but the pain persisted.

**Figure 1:**
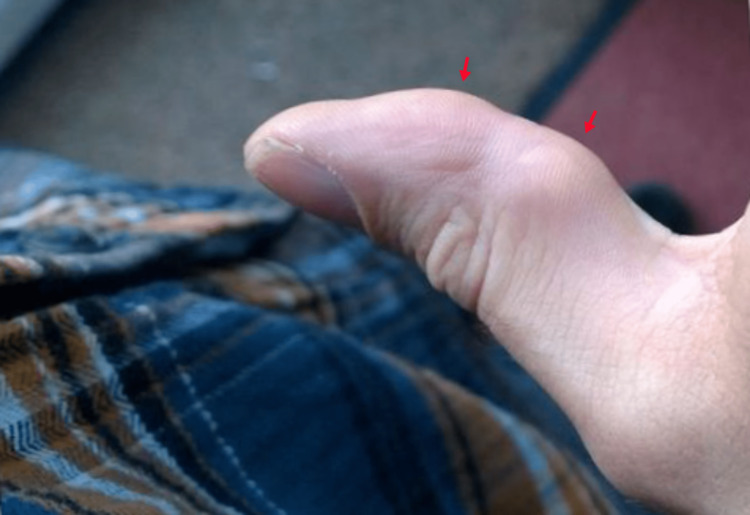
Right thumb showing multifocal swelling on the volar aspect

The patient had no significant medical history and was not taking any medications. On examination, the patient had two multifocal, non-tender subcutaneous swellings over the volar aspect of the right thumb at the first interphalangeal joint with a limited range of motion (Figure 1).

A moderate limitation in the range of motion was observed at the interphalangeal joint (IPJ), which may be attributed to the presence of swelling that is causing close contact between adjacent structures during full flexion. On the other hand, no abnormality was noted in the movement of the metacarpophalangeal joint (MCPJ). There was no paresthesia, and capillary circulation was normal. The radiographic evaluation of the hand demonstrated the presence of soft tissue edema located on the volar aspect of the thumb at the interphalangeal joint, and there was no evidence of any associated bone involvement.

**Figure 2:**
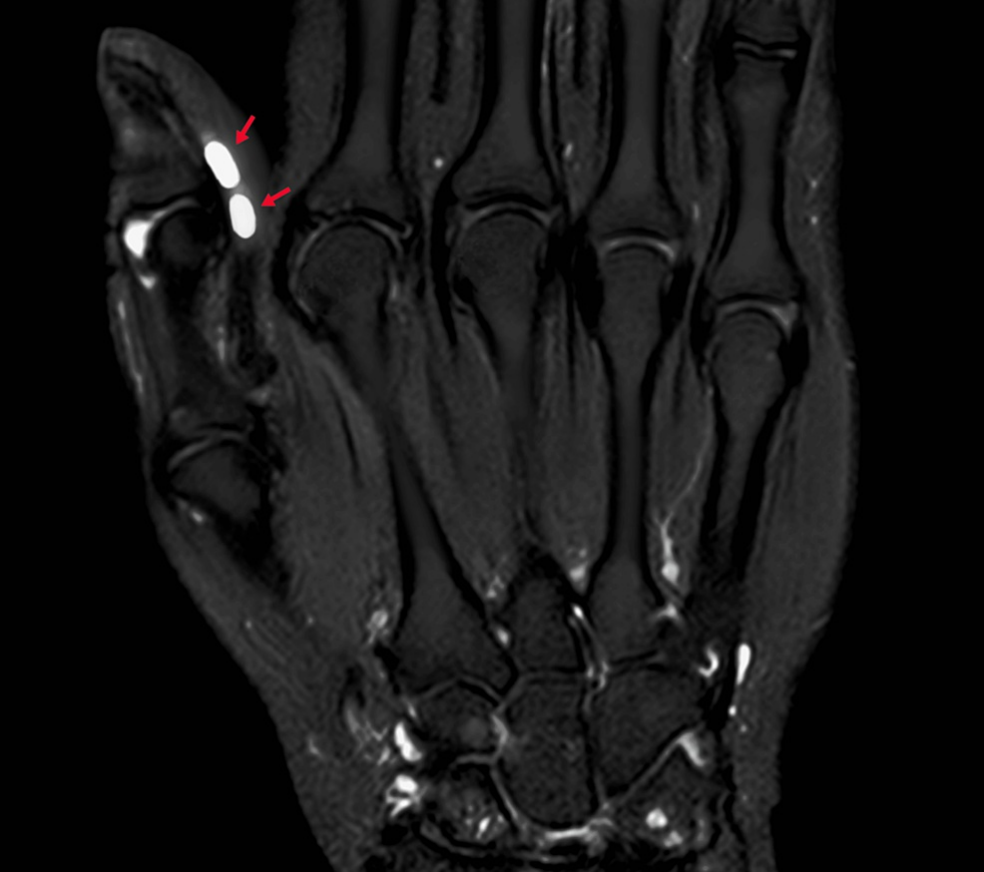
T2-image of the patient's right hand showing multifocal GCTTS along the FPL tendon (arrow) GCTTS: giant cell tumor of tendon sheath; FPL: flexor policies longus

The rest of the hand and wrist examination was normal. Magnetic resonance imaging (MRI) of the hand and wrist revealed two distinct swellings arising from the right thumb flexor pollicis longus (FPL) tendon. The sizes of the swellings were 0.3 x 0.2 cm and 0.2 x 0.2 cm from distal to proximal. These were iso-intense in T1-weighted images and hyper-intense in T2-weighted images (Figure 2).

**Figure 3:**
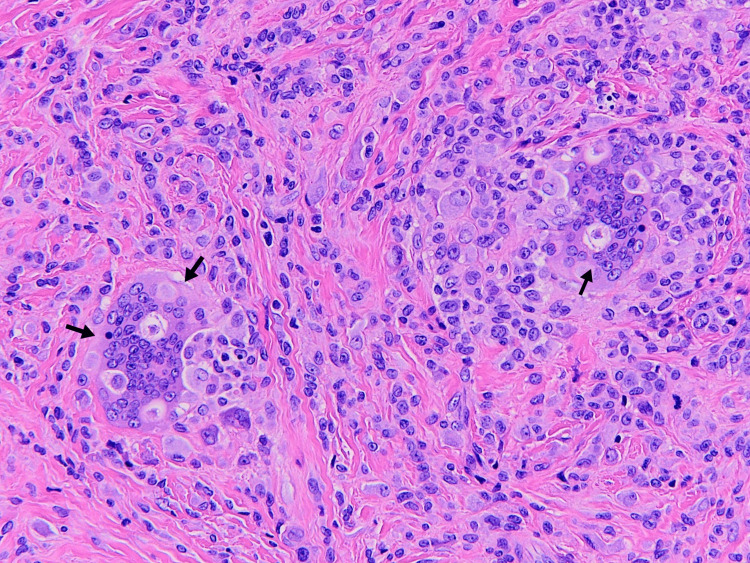
Multinucleated giant cells in a dense collagenous background (arrow)

Based on the clinical presentation and imaging studies, the patient was diagnosed with giant cell tenosynovitis (GCTTS) of the right thumb. A total excision of the swellings, a tenosynovectomy, was carried out by a team of surgeons under local anesthesia. Two isolated, firm, yellowish nodules were removed from the tendon of the FLP. The joint was not involved. The nodules were sent to the histopathology department for confirmation of the diagnosis of a giant cell tumor of the tendon sheath (GCTTS). The histopathological examination showed a dense collagenous background with xanthomatous cells, multinucleated giant cells, histiocytoid monocytes, and phagocytes within the collagen matrix, hence confirming our diagnosis of a localized benign form of GCTTS (Figure 3).

Postoperatively, a splint was applied to the thumb, and the wound healed completely without any infection. Two weeks after the procedure, the splint was removed, and the rehabilitation exercises for the thumb were advised to prevent any adhesion of the tendon. The patient's one, three, and six monthly follow-ups showed complete recovery with no radiological or clinical signs of any recurrence of GCTTS. His range of motion completely recovered, similar to his left thumb.

## Discussion

Giant cell tumors of the tendon sheath (GCTTS) are lesions that originate from the tendon sheath, joint synovium, or bursa and can present as either benign or locally aggressive. Depending on the nature of its presentation, GCTTS can be classified as either a localized (nodular tenosynovitis) or diffuse (pigmented villonodular synovitis (PVNS)) type. In the context of the hand, nodular tenosynovitis that is localized is the more common type, with the index finger being more frequently affected than the middle finger. Typically, this type of lesion arises on the volar aspect of the fingers, although it may also affect the dorsal aspect. [5]. The incidence rate is higher in males, with the mean age of patients being between 30 and 50 years. Typically, these tumors do not involve larger joints, and intra-articular placement is very rare [6].

The occurrence of multiple GCTTS lesions originating from the same tendon sheath is considered rare [1]. The first recorded case of multifocal GCTTS affecting a single tendon was documented in 2002 by Hitora et al., which described three nodules in the index finger [7]. Singh et al. also reported a case of multifocal GCTTS with five lesions, of which only two were noted before the surgical procedure while the remaining three were identified intraoperatively [8]. Additionally, Park et al. described a case involving a 33-year-old male with two separate lesions in the tendon of the flexor pollicis longus, with a gap of seven months between the appearance of the lesions [9]. Ansari et al. reported a case of four lobulated swellings arising from the flexor tendons of the index finger [1]. While none of these cases has experienced recurrence to date, the follow-up period for these patients has been less than two years. It is possible that in cases of multifocal GCTTS, the swelling may occur at different time intervals, which could be mistakenly identified as a recurrence when, in fact, they are new multifocal GCTTS arising from the same tendon [1].

The exact cause of GCTTS remains unknown and may be associated with various factors, including an inflammatory reaction, a disorder in local lipid metabolism, trauma-induced osteoclastic proliferation, or infection, among others [2]. Nevertheless, certain chromosomal alterations have been associated with the development of tenosynovial giant cell tumors, as reported by several studies. Due to the presence of a characteristic translocation, GCTTS is considered to be a clonal neoplastic lesion; it can recur locally and cause substantial morbidity, but metastases are rare [10]. Given that giant cell tumors lack distinctive clinical features, their diagnosis typically relies on investigations prompted by suspicion. Initial imaging often consists of plain radiographs, which typically reveal well-circumscribed areas of bone erosion. Cortical bone erosions are present in 10% to 14% of patients [6]. Ultrasonography can also be used to identify hypervascular areas of tumor and optimally guide synovial biopsy.

However, magnetic resonance imaging (MRI) is key to establishing the correct diagnosis. On magnetic resonance imaging (MRI), a distinctive absence of signal is observed on both T1 and T2 images, which has been linked to the abundance of hemosiderin in the synovial tissue [10].

In the present case, a diagnosis of GCTTS has been confirmed through both radiological and histopathological analysis. Upon histopathological examination, the tumor was found to be lobulated and encircled by a thick and transparent collagenous material. The tumor's intratumoral cells consisted of a combination of histiocytoid monocytes, multinucleated giant cells with an osteoblastic appearance, xanthoma cells, chronic inflammatory cells, ferruginous phagocytes, hemosiderin, and a matrix of collagen fibers [2]. The multinucleated cells express features of osteoclasts [10].

The choice of treatment is customized according to the patient's specific features, symptoms, the degree of advancement of the disease, anticipated postoperative complications, and the accessibility of systemic therapies. Patients with recurrent and/or relapsed disease may undergo observation or receive further surgical interventions, such as subtotal resection, joint replacement, or amputation. The primary approach to treating GCTTS is through local resection. While marginal excision is considered the ideal treatment for localized GCTTS, diffuse GCTTS presents a greater challenge in achieving complete eradication and is best managed with a total or near-total synovectomy. Studies have shown that the incidence of local recurrence is relatively low among patients with localized GCTTS, with approximately a 10% recurrence rate reported in one significant study. However, diffuse disease carries a higher risk of recurrence, ranging between 19% to 44% [10].

Furthermore, one of the studies has shown a recurrence rate of 10% to 20% after the procedure [6]. In our case, no recurrence was noted at the six-month follow-up.

## Conclusions

Giant cell tenosynovitis is a rare condition that presents as swelling over the affected tendon. Multifocal GCTTS is even rarer and can impair the range of motion at the joint, depending on its location. The diagnosis can be made based on clinical presentation and imaging studies. However, a histopathology examination is also important as it can further impact the management of the patient. With early diagnosis, management, and postoperative follow-up, most patients have a good prognosis with complete resolution of symptoms. Moreover, because of its high reoccurrence rates, the patient should be advised to follow up at regular intervals.
